# Development of an artificial intelligence‐based diagnostic model for Alzheimer's disease

**DOI:** 10.1002/agm2.12224

**Published:** 2022-09-25

**Authors:** Kazuki Fujita, Masahito Katsuki, Ai Takasu, Ayako Kitajima, Tomokazu Shimazu, Yuichi Maruki

**Affiliations:** ^1^ Department of Neurology Saitama Neuropsychiatric Institute Saitama City Saitama Japan; ^2^ Chichibu City Otaki National Health Insurance Clinic Chichibu Saitama Japan; ^3^ Department of Neurosurgery Itoigawa General Hospital Itoigawa Niigata Japan; ^4^ Department of Clinical Psychology Saitama Neuropsychiatric Institute Saitama City Saitama Japan

**Keywords:** Alzheimer's disease, artificial intelligence, dementia, diagnosis, primary health care

## Abstract

**Introduction:**

The diagnosis of Alzheimer's disease (AD) is sometimes difficult for nonspecialists, resulting in misdiagnosis. A missed diagnosis can lead to improper management and poor outcomes. Moreover, nonspecialists lack a simple diagnostic model with high accuracy for AD diagnosis.

**Methods:**

Randomly assigned data, including training data, of 6000 patients and test data of 1932 from 7932 patients who visited our memory clinic between 2009 and 2021 were introduced into the artificial intelligence (AI)‐based AD diagnostic model, which we had developed.

**Results:**

The AI‐based AD diagnostic model used age, sex, Hasegawa's Dementia Scale‐Revised, the Mini‐Mental State Examination, the educational level, and the voxel‐based specific regional analysis system for Alzheimer's disease (VSRAD) score. It had a sensitivity, specificity, and c‐static value of 0.954, 0.453, and 0.819, respectively. The other AI‐based model that did not use the VSRAD had a sensitivity, specificity, and c‐static value of 0.940, 0.504, and 0.817, respectively.

**Discussion:**

We created an AD diagnostic model with high sensitivity for AD diagnosis using only data acquired in daily clinical practice. By using these AI‐based models, nonspecialists could reduce missed diagnoses and contribute to the appropriate use of medical resources.

## Graphical abstract

In dementia medicine, the lack of accurate diagnostic tools for non‐specialists results in misdiagnoses and insufficient patient selection for a referral. We created an AI‐based diagnostic model with high sensitivity for AD diagnosis using only items acquired in daily clinical practice. Using these AI‐based models, non‐specialists can reduce misdiagnoses and contribute to starting the appropriate AD care and treatment.

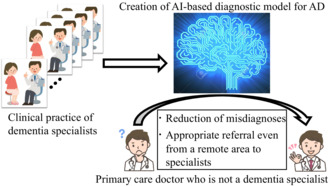

## 1 INTRODUCTION

Dementia is defined as a progressive decline in memory and impairment of executive functions. Dementia is a significant disease and causes a heavy burden for patients' health care providers.^1^ Currently, more than 50 million people worldwide live with dementia, and its prevalence increases exponentially with age; there are nearly 10 million new cases every year, according to a 2021 report by the World Health Organization.^2^ Moreover, cognitive decline after severe acute respiratory syndrome coronavirus 2 (SARS‐CoV‐2) infection has been widely reported,^3^ and the increase in the number of patients with dementia may accelerate.

An accurate diagnosis of dementia is the first step in addressing various problems of patients and caregivers; it provides guidance for appropriate management and predicts illness trajectory. Alzheimer's disease (AD), which is the most common form of dementia, may contribute to 60%–70% of cases of dementia.^2^ However, the prevalence of missed diagnosis in patients with dementia is also high.^4^ Additionally, in primary care settings, insufficient numbers of dementia specialists are a problem.^5^ By using diagnostic tools with high sensitivity, nonspecialists in areas with limited access to specialists can avoid overlooking AD. Appropriate screening for AD by nonspecialists contributes to earlier care and treatment. Ideal diagnostic models should consist of only simple items that nonspecialists can use during a consultation. Currently, the emerging field of artificial intelligence (AI) has been playing an increasingly crucial role in several aspects of medicine. Large clinical datasets of specialists' daily clinical practice can be used to construct AI‐based practical diagnostic tools, which may be helpful for primary care doctors. Although the exact process involved in the output of results remains unknown in AI, the proper usage of AI‐based tools can support clinical practice, especially for nonspecialists. The lack of access to dementia specialists and simple diagnostic tools for nonspecialists who treat people living in remote areas remains a problem. An accurate AI‐based AD diagnosis in the primary care setting can lead to rapid and appropriate referral and management to consulting specialists and may assist to predict illness outcomes. Therefore, in this study, we developed an AI‐based diagnostic model for AD.

## 2 METHODS

This retrospective study was conducted in our hospital from April 1, 2009, to March 30, 2021. The participants were outpatients who were admitted to our memory clinic and diagnosed with AD and other diseases as follows: (1) AD; (2) any dementia with Lewy bodies (DLB), frontotemporal dementia, vascular dementia, or other dementia; (3) mild cognitive impairment (MCI); (4) normal (absence of dementia); and (5) psychiatric disorders. AD was diagnosed according to the diagnostic criteria of the Diagnostic and Statistical Manual of Mental Disorders, 4th Edition. Patients with idiopathic normal‐pressure hydrocephalus, neurosurgical disorders, epilepsy, and neurodegenerative disorders except AD, DLB, and frontotemporal dementia were excluded.

We developed two AI‐based AD diagnosis models. First, seven independent variables were used to create the AI‐based diagnostic model, namely age, sex, Hasegawa's Dementia Scale‐Revised (HDS‐R), Mini‐Mental State Examination (MMSE), inability to perform the MMSE or HDS‐R, voxel‐based specific regional analysis system for Alzheimer's disease (VSRAD) score,^6^ and educational level. The HDS‐R is an evaluation tool for dementia that is widely used in Japan.^7^ The HDS‐R includes nine subdomains: (1) age, (2) time orientation, (3) space orientation, (4) immediate recall (registration), (5) calculation, (6) digits in reverse, (7) delayed recall, (8) item memory, and (9) speech fluency. The total score is 30 points, with a higher score indicating better cognitive ability. The cutoff for dementia is 20 points. Many studies have reported that the sensitivity of the HDS‐R (93%) is higher than that of the MMSE (82.8%), making it more suitable for AD screening.^8^ The educational level was divided into eight groups: elementary school, junior high school, high school, vocational school, junior college, university, graduate school, and others, such as schools for persons with hearing or communication impairments. After developing the model using the seven variables described above, we developed an AI‐based model without the VSRAD score, considering the low availability of the VSRAD score in rural areas.

We used the eXtreme Gradient Boosting (XGB) framework, Prediction One (Sony Network Communications, Inc.), to develop an AI‐based AD diagnostic model using the variables described above from the training dataset. The rationale behind the use of “Prediction One” is that it automatically performs preprocessing such as missing value completion and variable normalization, does not require the tuning of hyperparameters, and can be easily used by non‐AI engineers. After z‐normalization for each variable, we used XGB to classify the five diagnoses. According to previous reports, generalizability was ensured by fivefold cross‐validation.^9^, ^10^ Tuning was performed to increase the sensitivity. Precision, recall (same formula as sensitivity), *F* values, sensitivity, specificity, and *c*‐statics were used for model evaluation in the test dataset. The weight of each variable was investigated to determine how it contributed to the model reaching a diagnosis. We then performed backward variable selection to seek the best prediction model with small numbers of variables and high performance. The features were removed in order of decreasing weight. We also developed a prediction model using variables chosen by stepwise selection. To compare the usefulness of the models, we investigated the c‐statics (area under the curve; AUC) of the AI‐based models, HDS‐R, MMSE, VSRAD scores, and age for AD diagnosis.

The results are shown with mean ± standard deviation. We performed a *t* test for numerical variables and a chi‐square test for categorical variables and compared AUCs. The analysis was performed with SPSS Statistics 28.0.0 (IBM) and R software (Version 4.1.2). Significance was set at two‐tailed *P* < 0.05.

The ethics committee approved the study design. Informed consent was obtained in the form of opt‐out. All methods were performed according to the relevant guidelines and regulations of the Declaration of Helsinki.

## 3 RESULTS

### 3.1 Patient characteristics

A total of 8396 outpatients who visited our memory clinic and got diagnosed with AD; other dementias, MCI, and psychiatric disorders were screened for. Subsequently, 7932 were included in the analysis. Participants were randomly assigned to the training data of 6000 cases and test data of 1932 cases. The patient characteristics are shown in Table 1. The mean age was 77.43 years, and 62.5% were women. The mean MMSE and HDS‐R scores were 19.90 and 19.28, respectively. AD constituted 58.3% of dementia cases.

**TABLE 1:** Patient characteristics

	Total (*n* = 7932)	Training data (*n* = 6000)^a^	Test data (*n* = 1932)^a^	AD (*n* = 4630)	Non‐AD (*n* = 3302)
Diagnosis					
(1) AD	4630 (58.3%)	3466 (57.8%)	1164 (60.1%)	‐	‐
(2) Other dementia	1198 (15.1%)	901 (15.0%)	297 (15.3%)	‐	‐
(3) MCI	939 (11.8%)	708 (11.8%)	231 (11.0%)	‐	‐
(4) Normal	891 (11.2%)	701 (11.7%)	190 (9.8%)	‐	‐
(5) Psychiatric disorders	274 (3.5%)	206 (3.4%)	68 (3.5%)	‐	‐
Age	77.43 ± 9.71	77.28 ± 9.81	77.93 ± 9.83	80.02 ± 7.16*	74.15 ± 11.61*
Sex, female	4955 (62.5%)	3796 (63.2%)	1159 (59.8%)	3047 (65.8%)*	2111 (63.9%)*
Mean MMSE score	19.90 ± 6.31	19.90 ± 6.32	19.90 ± 6.37	17.46 ± 5.31*	22.98 ± 6.14*
Mean HDS‐R score	19.28 ± 7.18	19.30 ± 7.22	19.10 ± 7.29	16.29 ± 6.02*	22.92 ± 6.857*
Inability to perform MMSE or HDS‐R	32 (0.4%)	24 (0.4%)	8 (0.4%)	24 (0.52%)	12 (0.36%)
VSRAD Score	1.857 ± 2.12	1.842 ± 2.13	1.882 ± 2.22	2.183 ± 1.32	1.570 ± 2.77
Educational level					
Elementary school	431 (5.4%)	325 (5.4%)	106 (5.5%)	247	71
Junior high school	1755 (22.1%)	1309 (21.8%)	446 (23.0%)	849	459
High school	3252 (41.0%)	2491 (41.5%)	761 (39.2%)	1326	1084
Vocational school	747 (9.4%)	577 (9.6%)	170 (8.7%)	287	266
Junior college	141 (1.8%)	108 (1.8%)	33 (1.7%)	41	65
University	1567 (19.8%)	1159 (19.3%)	408 (21.1%)	518	656
Graduate school	38 (0.4%)	30 (0.5%)	8 (0.4%)	7	27
Other schools	1 (0.01%)	1 (0.01%)	0	0	2

Abbreviations: AD, Alzheimer's disease; HDS‐R, Hasegawa's Dementia Scale‐Revised; MCI, mild cognitive impairment; MMSE, mini‐mental state examination; VSRAD, voxel‐based specific regional analysis system for Alzheimer's Disease.

^a^ There was no statistically significant difference between the two groups.

*P < 0.001

### 3.2 AI‐based diagnostic model

The confusion matrix for the training and test data is shown in Tables 2 and 3. Table 2 shows the performance of Model 1 (consisting of seven variables), and Table 3 shows that of Model 2 (consisting of six variables exclusive of VSRAD).

**TABLE 2:** Confusion matrix for training and test data using seven variables

	Predicted diagnosis					
	(1) AD	(2) Other dementia^a^	(3) MCI	(4) Normal	(5) Psychiatric disorders	Recall
Training data						
True diagnosis						
(1) AD	3259	15	122	30	40	**0.9403**
(2) Other Dementia^a^	709	10	104	46	32	0.0111
(3) MCI	281	7	272	125	41	0.3747
(4) Normal	56	2	121	457	65	0.6519
(5) Psychiatric disorders	72	2	26	75	31	0.1505
Precision	**0.7466**	0.2788	0.4217	0.6235	0.1483	
Test data						
True diagnosis						
(1) AD	1111	2	29	8	14	**0.9544**
(2) Other dementia^a^	254	0	19	15	9	0
(3) MCI	102	1	59	36	15	0.2769
(4) Normal	33	0	16	120	21	0.6315
(5) Psychiatric disorders	31	1	5	20	11	0.1617
Precision	**0.7256**	0	0.4609	0.6030	0.1571	

*Note*: Recall and precision for AD diagnosis are described in bold.

^a^ Other dementias include Dementia with Lewy bodies, frontotemporal dementia, vascular dementia, and others.

Abbreviations: AD, Alzheimer's disease; MCI, mild cognitive impairment; Other dementias include Dementia with Lewy bodies, frontotemporal dementia, and vascular dementia.

**TABLE 3:** Confusion matrix for training and test data using six variables without VSRAD

	Predicted diagnosis					
	(1) AD	(2) Other dementia^a^	(3) MCI	(4) Normal	(5) Psychiatric disorders	Recall
Training data						
True diagnosis						
(1) AD	3243	11	138	37	37	**0.9357**
(2) Other Dementia^a^	714	17	94	42	44	0.0078
(3) MCI	296	1	264	117	48	0.3636
(4) Normal	65	1	111	439	85	0.6262
(5) Psychiatric disorders	75	2	28	67	34	0.1650
Precision	**0.7382**	0.3182	0.4157	0.6254	0.1371	
Test data						
True diagnosis						
(1) AD	1095	3	45	9	12	**0.9407**
(2) Other dementia^a^	249	2	23	17	6	0.0067
(3) MCI	83	1	77	34	18	0.3615
(4) Normal	19	0	29	126	16	0.6632
(5) Psychiatric disorders	30	0	7	21	10	0.1471
Precision	**0.7419**	0.3333	0.4254	0.6087	0.1613	

Abbreviations: AD, Alzheimer's disease; MCI, mild cognitive impairment; VSRAD, voxel‐based specific regional analysis system for Alzheimer's disease. The bold values mean recall and precision for an AD diagnosis in both training and test data.

^a^ Other dementias include Dementia with Lewy bodies, frontotemporal dementia, vascular dementia, and others.

Regarding Model 1, using seven variables in the training data, the precision, recall, *F*‐value, sensitivity, and specificity for AD were 0.7466, 0.9403, 0.8320, 0.9403, and 0.5568, respectively. These values in the test data were 0.7256, 0.9544, 0.8240, 0.9544, 0.5039, respectively. The weights that contributed to diagnostic accuracy are listed in Table 4. The MMSE, HDS‐R, and VSRAD scores contributed to AD diagnosis with high accuracy.

**TABLE 4:** Weights of each variable

Variables	Weight using seven variables	Weight using six variables
HDS‐R	1.330	1.510
MMSE	1.090	1.240
VSRAD score	0.998	NA
Age	0.847	0.914
Educational level	0.799	0.830
Sex	0.581	0.421
Inability to perform MMSE	0.415	0.401

Abbreviations: HDS‐R, Hasegawa's Dementia Scale‐Revised; MMSE, mini‐mental state examination; NA, not applicable; VSRAD, voxel‐based specific regional analysis system for Alzheimer's disease.

According to Model 2, using six variables exclusive of the VSRAD score in the training data, the precision, recall, F‐value, sensitivity, and specificity for AD were 0.7382, 0.9357, 0.8253, 0.9357, and 0.5479, respectively. For the test data, these were 0.7419, 0.9407, 0.8230,0.9407, and 0.4531, respectively. The MMSE, the HDS‐R, and age contributed to the diagnosis of AD with high accuracy (Table 4).

**FIGURE 1:**
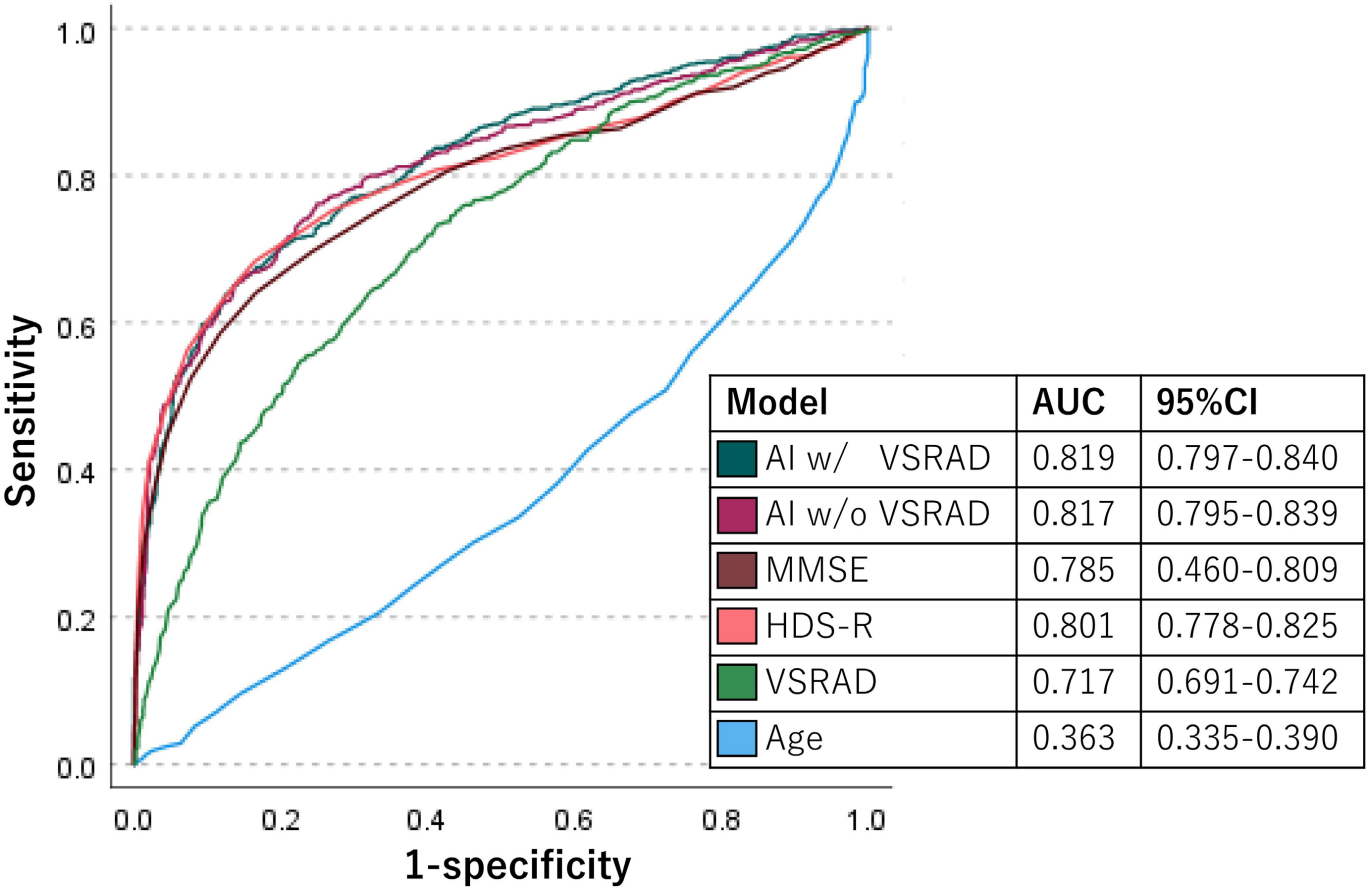
The area under the curve (AUC) is shown for each variable. The AUC of the AI‐based model with the VSRAD score and the AI‐based model without the VSRAD score, MMSE, HDS‐R, with the VSRAD score, and age were 0.819, 0.817, 0.785, 0.801, 0.717, and 0.363, respectively.

The area under the curve of the AI‐based model with the VSRAD and the AI‐based model without the VSRAD, MMSE, HDS‐R, VSRAD score, and age were 0.819, 0.817, 0.785, 0.801, 0.717, and 0.363, respectively (Figure 1). We compared the AUCs of AI with or without VSRAD score to HDS‐R alone. The *P* values were *P* = 0.0222 (AI with VSRAD score versus HDS‐R), *P* = 0.0253 (AI without VSRAD score versus HDS‐R), and *P* = 0.567 (AI with VSRAD score versus AI without VSRAD score), respectively. Therefore, the values of the AI‐based models were higher than those of the other scales and tests.

We performed backward and stepwise variable selection to seek the best prediction model with small numbers of variables and high performance. Table S1 shows the AUCs of the models created with variable selection. The model with all variables had the largest AUC.

## 4 DISCUSSION

We addressed two clinical issues. Our AI‐based diagnostic model has high precision and recall (sensitivity) for AD diagnosis, as shown in both the training and test datasets. This model enables nonspecialists to diagnose AD with a probability of more than 70% using only six or seven items with or without imaging evaluation. The most noteworthy point of this study is that the combination of simple information using an AI‐enabled diagnostic model detected not only the presence or absence of dementia but could also aid in the diagnosis of AD in the majority of cases.

The big data of memory clinic patients from our routine clinical practice produced an AI‐based diagnostic model with high precision and recall (sensitivity). Although novel drugs and biomarkers for early diagnosis in dementia medicine have been reported recently, there remains a considerable time gap before their clinical application. Under current conditions, optimizing dementia medicine in a primary care setting is essential. Primary care doctors generally engage in dementia medicine, regardless of their specialty. Hence, doctors are required to have some skills in dementia medicine. In this context, AI may change the existing practice of medicine.^11^ An advantage of AI is that it can make conventional methods of diagnosis and treatment more efficient, accurate, and effective. Although the weights of variables cannot fully explain how this impacted the results, the clinician's appropriate use of an AI‐based model can support a clinical decision in a reliable form.

Past studies have reported inadequate diagnostic accuracy for dementia in primary care settings. The MMSE is often used in primary care settings and is the most thoroughly studied instrument. A systematic review of 14 studies (10,185 participants) resulted in a sensitivity and specificity of 88.3% and 86.2%, respectively, for a cutoff point of 23/24 or 24/25 to detect dementia.^12^ However, this is limited to the diagnosis of dementia without other types of dementia. The HDS‐R is the most used evaluation tool for dementia in Japan, equivalent to the MMSE.^7^ The results of the current study matched those of previous HDS‐R studies on brain atrophy, which suggests a relationship between HDS‐R results and hippocampal atrophy.^13^ A recently shortened HDS‐R was reported to be similar to the full HDS‐R.^8^

In this study, the AD diagnostic tool used seven items. These items matched the specialists' clinical abilities based on their years of experience. Moreover, the HDS‐R, MMSE, VSRAD score, age, and educational level contributed to AD diagnosis in the same order. First, the HDS‐R lacks cutoffs modified by the participants' age and educational level. Older age and lower educational levels usually increase the probability of developing AD. A previous study provided an appropriate cutoff, which was adjusted by age and educational level.^14^ Our study might compensate for this weak point of the daily use of the HDS‐R. Our results match those of a recent report describing the risk of low education.^15^ Second, the VSRAD score showed an association with a clinical diagnosis of dementia. Therefore, most doctors use the VSRAD score to confirm a clinical diagnosis. The VSRAD score reflects age‐related atrophy of the brain during its developmental process.^6^ Our study may indirectly ensure this process.

Moreover, this diagnostic model can be used in other clinical settings. Our tool contains common questions that can be obtained in routine clinical practice. Our AI‐based model can enable patients living in rural areas with no dementia specialists and without head radiological imaging to be appropriately screened with accuracy so that the next steps can be decided. A significant problem in dementia management is the imbalance between the increasing number of patients and the limited number of doctors who are specialists in dementia.^5^ It is not realistic to expect that all patients suspected of having dementia by their primary care doctors will be referred to dementia specialists. Access to specialists in dementia is difficult for people living in remote areas and islands. Additionally, patients with dementia who visited primary care doctors were reported to have an increased need for postdiagnostic support than those who visited specialists.^16^

Although we tried to make an AI‐based prediction model detect cases that convert from MCI to AD like eye‐tracking technology,^17^ our database was insufficient to develop such a model. Later, we developed the AD diagnostic tool to solve the current unmet needs. In the future, AI diagnostic support can result in enhancing the quality of dementia medicine and can be widely used as a communication tool between non‐specialists and specialists. Uniting specialists and family physicians is an efficacious option to address these problems.^18^ In Japan, having “dementia support doctors” who support both primary care doctors and dementia specialists has been encouraged as an alternative to increasing the number of dementia specialists alone.^19^ To stabilize this movement, a diagnostic tool that can be used in the primary care setting is warranted. However, highly accurate AD diagnostic tools have not been reported to date. In order to address this need for a highly accurate AD diagnostic tool, we developed our AI‐based AD diagnostic model. This model has the potential to solve the problem of low levels of access to dementia experts in a population where the number of dementia patients is increasing. Furthermore, AI‐based support can be used as a screening tool in telemedicine, which may suggest an appropriate time to get an imaging evaluation. This support can reduce the burden of a hospital visit and contribute to improvement in health economics. AI analysis of progressive changes in dementia may enable the prediction of the prognosis; adding an AI‐embedded tool to a conventional medical questionnaire may open up the possibility of personalized medicine in order to predict the disease trajectory more accurately.

The limitation of this study was that we used no biomarkers to determine the pathological changes of AD. Although a pathologically accurate analysis is ideal, we thought that practical usefulness was more critical than pathologic confirmation of AD. Second, all participants were from a single hospital. This diagnostic model can be a reliable diagnostic tool, provided that it is validated in other cohorts. Third, cranial imaging is required in some cases to exclude secondary dementia. However, clinicians, even nonspecialists in dementia, can usually identify these cases through appropriate history taking and physical examination. Fourth, clinicians should diagnose early‐onset AD using a combination of methods rather than relying solely upon our diagnostic tools. Patients with early‐onset AD often maintain their scores on neuropsychological tests.

## 5 CONCLUSION

This study created an AI‐based tool to diagnose AD in situations with limited resources, bearing in mind that a simple diagnostic tool for MCI is warranted in the future to detect cognitive decline at an earlier stage.

We created an AD diagnostic model with high precision and recall (sensitivity) using only the items acquired during a consultation, which nonspecialists can implement quickly into their daily clinical practice as a practical diagnostic. Through the use of this diagnostic model, an AD diagnosis by nonspecialists may reduce misdiagnosis and contribute to the appropriate and timely use of medical resources.

## AUTHOR CONTRIBUTIONS

Kazuki Fujita wrote the main manuscript text and edited the manuscript. Masahito Katsuki was responsible for statistical analysis and artificial intelligence development. Ai Takasu and Ayako Kitajima contributed to the data collection. Dr. Tomokazu Shimazu and Yuichi Maruki supervised the manuscript. All authors reviewed and approved the final manuscript.

## FUNDING INFORMATION

The authors have no sources of funding to declare.

## CONFLICT OF INTEREST

The authors report no conflicts of interest for this study.

## ETHICS APPROVAL

Ethics Committee issued approval 2021‐11‐1.

## PATIENT CONSENT

Informed consent was obtained in the form of opt‐out.

## Supporting information


Table S1

## Data Availability

The data supporting this study's findings are available from the corresponding author upon reasonable request.

## References

[1] Feigin VL , Vos T , Nichols E , et al. The global burden of neurological disorders: translating evidence into policy. Lancet Neurol. 2020;19(3):255‐265. doi:10.1016/S1474-4422(19)30411-9 31813850 PMC9945815

[2] World Health Organization . Global action plan on the public health response to dementia 2017–2025. Accessed August 31, 2022. https://apps.who.int/iris/handle/10665/259615

[3] Liu YH , Chen Y , Wang QH , et al. One‐year trajectory of cognitive changes in older survivors of COVID‐19 in Wuhan, China: a longitudinal cohort study. JAMA Neurol. 2022;79(5):509‐517. doi:10.1001/jamaneurol.2022.0461 35258587 PMC8905512

[4] Skinner TR , Scott IA , Martin JH . Diagnostic errors in older patients: a systematic review of incidence and potential causes in seven prevalent diseases. Int J Gen Med. 2016;9:137‐146. doi:10.2147/IJGM.S96741 27284262 PMC4881921

[5] Hum S , Cohen C , Persaud M , et al. Role expectations in dementia care among family physicians and specialists. Can Geriatr J. 2014;17(3):95‐102. doi:10.5770/cgj.17.110 25232368 PMC4164682

[6] Matsuda H . Voxel‐based morphometry of brain MRI in normal aging and Alzheimer's disease. Aging Dis. 2013;4(1):29‐37. 23423504 PMC3570139

[7] Katoh S , Simogaki H , Onodera A , et al. Development of the revised version of Hasegawa's Dementia Scale (HDS‐R). Jpn J Geriatr Psychiatry. 1991;2(11):1339‐1347. (in Japanese).

[8] Gong Q , Ishii M , Numata O , Xie W , Hirata T . Utility of a shortened Hasegawa dementia scale revised questionnaire to rapidly screen and diagnose Alzheimer's disease. Aging Med (Milton). 2021;2:109‐114. doi:10.1002/agm2.12152 PMC8251873 34250428

[9] Maki S , Furuya T , Yoshii T , et al. Machine learning approach in predicting clinically significant improvements after surgery in patients with cervical ossification of the posterior longitudinal ligament. Spine. 2021;46(24):1683‐1689. doi:10.1097/BRS.0000000000004125 34027925

[10] Katsuki M , Kakizawa Y , Nishikawa A , Yamamoto Y , Uchiyama T . Easily created prediction model using deep learning software (Prediction One, Sony Network Communications Inc.) for subarachnoid hemorrhage outcomes from small dataset at admission. Surg Neurol Int. 2020;11:374. doi:10.25259/SNI_636_2020 33408908 PMC7771510

[11] Amisha MP , Pathania M , Rathaur VK . Overview of artificial intelligence in medicine. J Family Med Prim Care. 2019;8(7):2328‐2331. doi:10.4103/jfmpc.jfmpc_440_19 31463251 PMC6691444

[12] Lin JS , O'Connor E , Rossom RC , et al. Screening for Cognitive Impairment in Older Adults: An Evidence Update for the U.S. Preventive Services Task Force. Agency for Healthcare Research and Quality (US); 2013. 24354019

[13] Bizen H , Kimura D , Ohtoshi T . Relationship among hippocampal atrophy, brain blood flow, and neuropsychological tests in patients who experienced forgetfulness. J Allied Health Sci. 2017;8(2):104‐109. (in Japanese).

[14] Kim KW , Lee DY , Jhoo JH , et al. Diagnostic accuracy of mini‐mental status examination and revised hasegawa dementia scale for Alzheimer's disease. Dement Griatr Cogn Disord. 2005;19(5–6):324‐330. doi:10.1159/000084558 15785033

[15] Livingston G , Huntley J , Sommerlad A , et al. Dementia prevention, intervention, and care: 2020 report of the Lancet Commission. Lancet. 2020;396(10248):413‐446. doi:10.1016/S0140-6736(20)30367-6 32738937 PMC7392084

[16] Michelet M , Lund A , Strand BH , Engedal K , Selbaek G , Bergh S . Characteristics of patients assessed for cognitive decline in primary healthcare, compared to patients assessed in specialist healthcare. Scand J Prim Health Care. 2020;38(2):107‐116. doi:10.1080/02813432.2020.1753334 32362213 PMC8570739

[17] Oyama A , Takeda S , Ito Y , et al. Novel method for rapid assessment of cognitive impairment using high‐performance eye‐tracking technology. Sci Rep. 2019;9(1):12932. doi:10.1038/s41598-019-49275-x 31506486 PMC6736938

[18] Lee L , Kasperski MJ , Weston WW . Building capacity for dementia care: training program to develop primary care memory clinics. Can Fam Physician. 2011;57(7):e249‐e252. 21753083 PMC3135463

[19] Washimi Y , Horibe K , Takeda A , Abe T , Toba K . Educational program in Japan for dementia support doctors who support medical and care systems as liaisons for demented older adults in the community. Geriatr Gerontol Int. 2014;14(Suppl 2):11‐16. doi:10.1111/ggi.12248 24650060

