# Supplementary material for: Development of an artificial intelligence‐based diagnostic model for Alzheimer's disease
Source: Aging Med (Milton). 2022 Sep 25;5(3):167–73. doi: 10.1002/agm2.12224 (PMC9549305; doi:10.1002/agm2.12224)
Supplement: Supplementary file 1 — Table S1 [file AGM2-5-167-s001.docx]

**Supplementary Table 1. Feature selection**

| **Variables used for AI model development** | **c-statistics†** |
| --- | --- |
| HDS-R, MMSE, VSRAD Score, Age, Educational level, Sex, Inability to perform MMSE | 0.8192 |
| HDS-R, MMSE, VSRAD Score, Age, Educational level, Sex | 0.8174 |
| HDS-R, MMSE, VSRAD Score, Age, Educational level | 0.8136 |
| HDS-R, MMSE, VSRAD Score, Age | 0.8098 |
| HDS-R, MMSE, VSRAD Score | 0.8091 |
| HDS-R, MMSE | 0.7894 |
| HDS-R, Age, Educational level, Sex | 0.7876 |

Abbreviations: HDS-R, Hasegawa’s Dementia Scale-Revised; MMSE, mini-mental state examination; VSRAD, voxel-based specific regional analysis system for Alzheimer’s disease; NA, not applicable. †; Feature (variable) selection was performed a based-on backward selection. The features were removed in order of decreasing weight.
